# Genome-wide association study for carcass weight in pasture-finished beef cattle in Hawai’i

**DOI:** 10.3389/fgene.2023.1168150

**Published:** 2023-05-09

**Authors:** Mandeep Adhikari, Michael B. Kantar, Ryan J. Longman, C. N. Lee, Melelani Oshiro, Kyle Caires, Yanghua He

**Affiliations:** ^1^ Department of Molecular Biosciences and Bioengineering, University of Hawai’i at Mānoa, Honolulu, HI, United States; ^2^ Department of Tropical Plant and Soil Sciences, University of Hawai’i at Mānoa, Honolulu, HI, United States; ^3^ East West Center, Honolulu, HI, United States; ^4^ Department of Geography and Environment, University of Hawai’i at Mānoa, Honolulu, HI, United States; ^5^ Department of Human Nutrition, Food, and Animal Sciences, University of Hawai’i at Mānoa, Honolulu, HI, United States

**Keywords:** GWAS, pasture-finished beef, carcass weight, Hawai’i, SNPs

## Abstract

**Introduction:** Genome-wide association studies (GWAS) have identified genetic markers for cattle production and reproduction traits. Several publications have reported Single Nucleotide Polymorphisms (SNPs) for carcass-related traits in cattle, but these studies were rarely conducted in pasture-finished beef cattle. Hawai’i, however, has a diverse climate, and 100% of its beef cattle are pasture-fed.

**Methods:** Blood samples were collected from 400 cattle raised in Hawai’i islands at the commercial harvest facility. Genomic DNA was isolated, and 352 high-quality samples were genotyped using the Neogen GGP Bovine 100 K BeadChip. SNPs that did not meet the quality control standards were removed using PLINK 1.9, and 85 k high-quality SNPs from 351 cattle were used for association mapping with carcass weight using GAPIT (Version 3.0) in R 4.2. Four models were used for the GWAS analysis: General Linear Model (GLM), the Mixed Linear Model (MLM), the Fixed and Random Model Circulating Probability Unification (FarmCPU), the Bayesian-Information and Linkage-Disequilibrium Iteratively Nested Keyway (BLINK).

**Results and Discussion:** Our results indicated that the two multi-locus models, FarmCPU and BLINK, outperformed single-locus models, GLM and MLM, in beef herds in this study. Specifically, five significant SNPs were identified using FarmCPU, while BLINK and GLM each identified the other three. Also, three of these eleven SNPs (“BTA-40510-no-rs”, “BovineHD1400006853”, and “BovineHD2100020346”) were shared by multiple models. The significant SNPs were mapped to genes such as *EIF5*, *RGS20*, *TCEA1*, *LYPLA1*, and *MRPL15*, which were previously reported to be associated with carcass-related traits, growth, and feed intake in several tropical cattle breeds. This confirms that the genes identified in this study could be candidate genes for carcass weight in pasture-fed beef cattle and can be selected for further breeding programs to improve the carcass yield and productivity of pasture-finished beef cattle in Hawai’i and beyond.

## Introduction

In Hawai’i, there is a considerable amount of land classified as pasture, much of which is suitable grazing land (Fukumoto et al., 2015). Year-round availability of forage favors cattle production and the beef industry in Hawai’i which significantly contributes to the state’s economy (Asem-Hiablie et al., 2018). In Hawai’i there are two distinct climatic seasons: The relatively hot dry season runs from May to October, and the cool wet season runs from November to April (Giambelluca et al., 2014). Despite differences in the season, the tropical location of the Islands provides ample sunlight and moisture that supports year-round forage growth necessary for pasture-finished cattle farming which is less common in the continental United States and other regions. However, cattle stocking rates are often decreased during the dry season due to limited forage growth (Adhikari et al., 2022). At present, the total pasture area in the state is about 448,513 hectares, most of which (> 50%) is located on the Island of Hawai’i (Melrose et al., 2015). The limited availability of land is the main constraint for pasture-finished beef production in Hawai’i. Approximately 85% of calves are exported to the continental United States at weaning age and only 15% remain for local food supplies, which satisfied only 10%–13% of the local meat demand, and the gap is fulfilled by the imported meat (Asem-Hiablie et al., 2018; National Agricultural Statistics Service, USDA, 2021). There is a growing demand for pasture-finished beef among tourists and local consumers in Hawai’i, and genetic improvement of beef cattle is necessary for Hawai’i to increase production and improve productivity, leading to a larger local supply.

Carcass weight is a critical factor that affects beef cattle production and its economic returns (S.-H. Lee et al., 2014). This trait is impacted by both genetic and environmental factors (Irshad et al., 2013). Despite its significance, a limited number of studies have been conducted on Hawai’i cattle. A survey by Asem-Hiablie et al. (2018) explored the management practices, herd size, feeding, and marketing strategies under various production systems. Fukumoto and Kim (2007) examined carcass characteristics of forage-finished cattle in Hawai’i. Additionally, pasture-finished beef from Hawai’i competes with feed-lot-finished beef from the continental United States in terms of tenderness and marbling score (Kim, Fukumoto, and Kim, 2012). Information on cattle genetics and dedicated research on genes governing carcass yield and meat quality is an emerging scientific field that at present is under study in Hawai’i.

Genetic interventions hold the potential to significantly boost net carcass production and expand the local supply without additional retention of cattle heads for finishing. With the development of genetic testing technologies and the reduction in the cost of genotyping, significant advances in research have been made over the past two decades to improve the breeding and genetics of domestic cattle (Van Tassell et al., 2008; Weller et al., 2017; Tam et al., 2019). Affordable genotyping cost and the tendency of single nucleotide polymorphism (SNPs) to follow a pattern of linkage disequilibrium (LD) across the genome has opened up the arena for several genomic studies such as ancestry analysis, genomic selection (GS) and genome-wide association studies (GWAS) (Gautier et al., 2010; Goddard and Hayes, 2012; Decker et al., 2014). SNPs have been efficiently used to evaluate economic traits in cattle, such as carcass weight (Rolf et al., 2012; Costa et al., 2015; Keogh et al., 2021). Using SNP markers to evaluate cattle production and productivity is becoming essential in commercial cattle farming (Smith et al., 2019; Keogh et al., 2021). Hawai’i cattle, which are raised exclusively on pasture and thrive in the diverse geography and environment of the state, may possess unique genetic markers for carcass weight. Previous studies have successfully reported SNPs related to carcass weight, growth traits, feed intake, environmental adaptation, and meat quality traits in grain-finished or grass-finished production systems (Rolf et al., 2012; Costa et al., 2015; Silva et al., 2017; Smith et al., 2019; Keogh et al., 2021). However, there is a shortage of research on genetic markers, candidate genes, and their expression in pasture-finished beef in the tropical Pacific environment, and no specific genetic markers have been identified for Hawai’i cattle. (S. H. Lee et al., 2013; Silva et al., 2017; Edea et al., 2018; Hay and Roberts, 2018). Thus, focused genome-wide association studies (GWAS) in Hawai’i cattle can be valuable in discovering unique markers or verifying existing SNP markers for pasture-finished beef cattle in the tropical environment of Hawai’i.

One of the main challenges for the GWAS analysis is managing false positives and false negatives that may occur due to population structure and familial relationships. To address this issue, mixed linear models (MLMs) are commonly used, incorporating covariates for structure and kinship to control for false positives. LD is the non-random association of SNPs markers at different chromosome loci and is mainly determined by the physical distance between the markers, which can influence false positive and false negative rates. Several factors, such as population stratification, migration, recombination, mutation, and selection, affect the pattern of LD in a population (Goddard and Hayes, 2012; Karimi et al., 2015; Singh et al., 2021). In this study, four different statistical models, were compared for GWAS analysis for carcass weight in Hawaiian beef herds, including single-locus models: GLM—General Linear Model and MLM—Mixed Linear Model, and multi-locus models: FarmCPU and BLINK (Huang et al., 2019; Miao et al., 2019). GLM uses population structure (Q) as a covariate, and MLM uses both population structure and kinship (Q + K) as covariates. The FarmCPU model consists of the fixed-effect model (FEM) and the random-effect model (REM), which is evaluated iteratively. The effects in the FEM include the significant principal components, sex, and pseudo-quantitative trait nucleotides (Liu et al., 2016; Tang et al., 2019). Unlike GLM and MLM, which rely on one-to-one markers and trait correlation, FarmCPU trains multiple markers and builds correlations with significant SNP markers simultaneously. Additionally, markers from other loci are utilized as covariates to partially eliminate the confounding effects of the markers and kinship (Liu et al., 2016). BLINK is an enhanced version of FarmCPU (Wang and Zhang, 2021), which replaced REM with FEM for the model selection of the pseudo QTNs. Consequently, the iterations are eliminated to optimize the genetic-to-residual variance ratio, generating a higher statistical power than FarmCPU (Liu et al., 2016; Huang et al., 2019).

Therefore, this study aims to test several statistical models and determine the most appropriate model for association mapping in Hawai’i beef cattle by using carcass weight as a phenotype. The main objective of this paper is to identify SNP markers and candidate genes related to carcass weight for pasture-finished beef cattle. The goal of this research is to advance the genetic enhancement of beef cattle by incorporating the identified candidate genes in breeding programs in Hawai’i.

## Methodology

### Sample collection and genotyping

**FIGURE 1:**
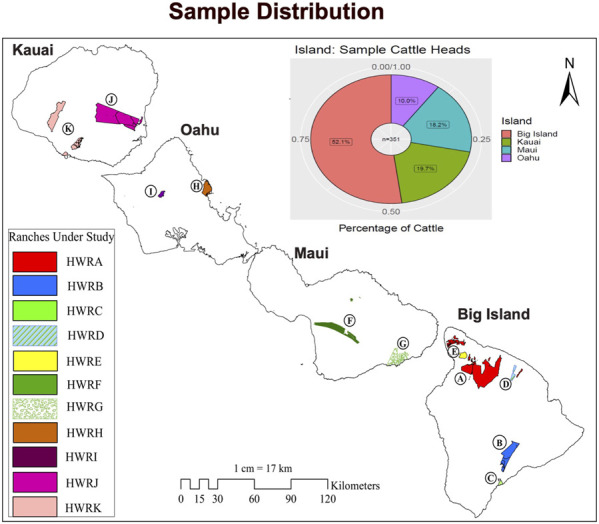
The cattle in this study and their distribution on Hawaiian islands. Eleven Hawaiian ranches coded as HWRA-HWRK are highlighted on the map of Hawai’i. The pie chart represents the percentage of cattle heads (*N* = 351) in each island (Big Island, Maui, Oahu, and Kauai) sampled in this study.

Samples were collected from 400 beef cattle at the commercial harvest facility in Kapolei, on the Island of Oahu. The sampling was random and included representatives from the major eleven ranches on four Islands in Hawaii: the Island of Hawaii (Big Island), Maui, Oahu, and Kauai. Both male and female cattle were included, and the age of the cattle ranged from younger than 30 months to older than 30 months to ensure that the samples were truly representative of the Hawai’i cattle population. The original source farms for the samples are mapped in the base map of Hawaii. However, to maintain the confidentiality of the commercial farms, we assigned them a nickname with Alphabet A to K, such as Hawaii Ranch A (HWRA) and Hawaii Ranch B (HWRB), as shown in Figure 1. All details about the source farms’ location in the island chain and the number of cattle heads sampled from each farm can be found in Supplementary Table S1. At the slaughter plant, 10 mL of whole blood sample was taken from the jugular vein of each cattle using EDTA anticoagulant tubes, placed on ice, and eventually brought back to the laboratory where it was stored at −80°C. Genomic DNA was isolated from the blood samples using Quick-DNA Miniprep Plus Kit (Zymo Research, D4069), followed by the concentration measurement using NanoDrop™ One Microvolume UV-Vis Spectrophotometers. A total of 352 DNA samples with a concentration higher than 100 ng/uL and OD values between 1.8 and 2.2 were loaded for genotyping using the Neogen GGP Bovine 100 K BeadChip with the ARS-UCD1.2 assembly (Rosen et al., 2020). The raw genotypic data were checked and filtered for missing genotypes greater than 10%, minor allele frequencies (MAF) less than 0.05, Hardy Weinberg Equilibrium *p*-value less than 10^–6^, and missingness per individual greater than 10%. SNPs on mitochondrial DNA and sex chromosomes were removed using PLINK 1.9. Therefore, 85 K SNPs in 351 cattle after the quality control were used in further analysis to identify the genetic markers associated with carcass weight in Hawai’i beef cattle herds. All animal experiments were conducted in accordance with NIH guidelines for housing and care of laboratory animals and under the University of Hawaii (UH) regulations UH IACUC Policy 18.0 after review and approval by the UH Animal Welfare and Biosafety Programs Committee (Assurance number A3423-01).

### GWAS analysis

To test for normality, the Shapiro test was performed on the phenotypic data, specifically the carcass weight. Since the original data are not normally distributed, a log transformation was performed to normalize it. The normalized data on carcass weight were then subjected to a one-way analysis of variance (ANOVA), followed by *post hoc* Fisher’s least significant difference (LSD) test using the Agricolae package (Steel, 1997) in R 4.2 (https://www.R-project.org/). Statistical significance was evaluated using an alpha value of 0.05, and any relationships falling below this value were considered significant. The log-transformed phenotypic data were eventually used in the GWAS analysis. GAPIT (version 3.0) R package (Wang and Zhang, 2021) was used to conduct association mapping with four different models: i) General Linear Model (GLM) (Price et al., 2006), ii) Mixed Linear Model (MLM) (Yu et al., 2006), iii) Fixed and random model circulating probability unification (FarmCPU) (Liu et al., 2016), and iv) Bayesian-information and Linkage-disequilibrium Iteratively Nested Keyway (BLINK) (Huang et al., 2019). Carcass weight was the phenotype to be tested, where age, sex, and farms were incorporated as covariates in all models to avoid confounding effects. All other parameters were set as default. GAPIT comes with a built-in function for intermediate analysis, which includes principal component (PC) analysis, kinship matrix calculation, and linkage disequilibrium decay (LD). To compute the kinship matrix, the algorithm developed by VanRaden (2008) was used. The developer team of GAPIT has provided a user-friendly manual (https://zzlab.net/GAPIT/gapit_help_document.pdf) that includes fundamental codes and pipelines with a brief explanation of the models and algorithms used for association mapping. The pipeline was followed in our study, and the LD decay plot was displayed over distance from the LD results from TASSEL 5 using R software. To check our population stratification, the genomic inflation factor, Lambda (λgc), was estimated, which is determined by comparing the median of the chi-squared test statistics obtained from a GWAS to the anticipated median of the chi-squared distribution. The median value of a chi-squared distribution with one degree of freedom is 0.4549364. The approach used to calculate lambda can differ based on the association analysis output, such as z-scores, chi-square statistics, or *p*-values (Devlin and Roeder, 1999)**.** In this study, we used *p*-values from the GWAS outcomes of all four models (GLM, MLM, FarmCPU, and BLINK) and computed λgc using the *qchisq*() function in R Studio 4.2. To correct for population structure, the first PC was fitted in the model, and the kinship matrix was added to account for the confounding effect of ancestry. Marker-trait associations were considered significant if they met an exploratory threshold of *p* < 10–6 (-log10*p* > 6), which were displayed in Manhattan plots. The validity of associations was verified using the quantile-quantile (QQ) plot to distinguish between true and spurious results, such as false positives and false negatives (Stich et al., 2008; Kristensen et al., 2018). Additionally, the *p*-value threshold was adjusted for False Discovery Rate (FDR < 0.05), and any additional SNPs were listed in Supplementary Table S2.

### Candidate gene selection and functional annotations

The result of linkage disequilibrium decay (LD) was used as a sliding window to find the genes within a certain distance from the identified SNP markers using the latest reference genome assembly for cows (*bosTau9 or ARS-UCD 1.2 genome assembly*) (Rosen et al., 2020). Candidate genes were selected within 100 kb upstream and downstream of the significant SNPs based on LD value for our cattle population. Three major genome browsers, UCSC (https://genome.ucsc.edu/), NCBI (https://www.ncbi.nlm.nih.gov/), and Ensembl (https://uswest.ensembl.org/index.html), were used to annotate the significant SNPs identified for carcass weight in Hawai’i cattle population. The function of the candidate genes, their homologs, and their roles in carcass-related traits and other mammals were explored through GeneCards (http://www.genecards.org/) and UniProt/Swiss-Prot browser (http://uniprot.org).

## Results

### Population distribution and phenotypic analysis

The population in our study included cows, steers, and heifers (*N* = 351 cattle heads) from eleven diversified farms located across the Islands of Hawaii (Big Island), Maui, Oahu, and Kauai (Figure 1). Over 52% of the cattle in this study are located on the Big Island, and the remaining 48% of the cattle were from neighboring islands, with at least two farms from each island. As reported, Hawaii and Maui have the largest cattle herds, contributing to more than 70% of the cattle heads in the Hawai’i island chain, followed by Kauai and Oahu (National Agricultural Statistics Service, USDA, 2021). Most of the ranching activities in Hawai’i were concentrated on the Big Island and Maui due to the availability of suitable pasture and their favorable climate for forage growth. In our study, 70% of the cattle heads were taken from these two islands (Figure 1), making our sample population proportionately represent the beef cattle distribution in Hawai’i.

**FIGURE 2:**
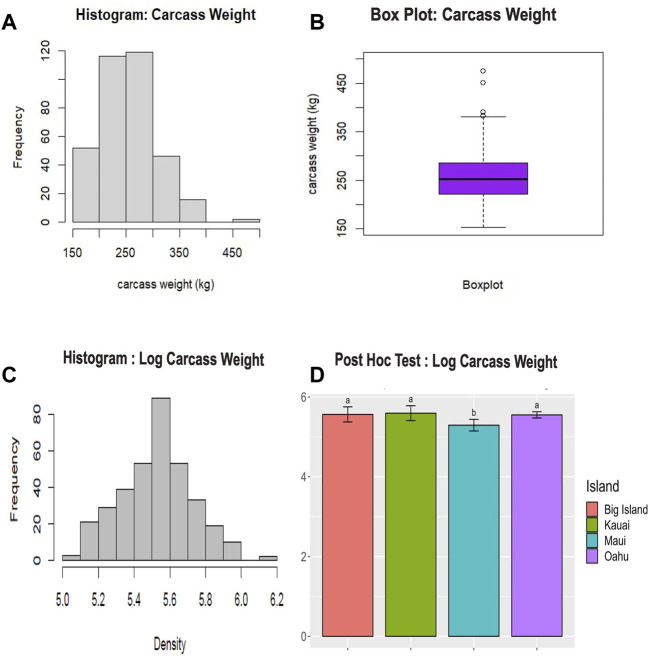
Phenotypic data analysis for carcass weight of cattle. **(A)** The histogram displays the distribution of unadjusted carcass weight. **(B)** The boxplot summarizes the statistical information of the unadjusted carcass weight, including outliers. **(C)** The histogram shows the distribution of log-transformed carcass weight. **(D)** Fisher’s *post hoc* least significant difference (LSD) test (*p < 0.05*) conducted across the islands. This *post hoc* test followed an Analysis of Variance (ANOVA) test *(p < 0.05*). The lowercase letters above the bar plot indicate significant differences among the groups. Groups with the same letter are not significantly different from each other, while groups with different letters are significantly different.

The raw phenotypic data for carcass weight failed the Shapiro test for normality (*p < 0.05*), indicating a deviation from a normal distribution. The results of the raw data revealed a right-skewed distribution with a few extreme values (Figures 2A, B). Thus, we log-transformed the raw data before conducting further analysis. The normality test on transformed data showed a normal distribution (*p > 0.05*), with the majority of the data concentrated around the central value (Figure 2C). Analysis of variance conducted on the log-transformed carcass values demonstrated a significant difference (*p < 0.05*) in carcass yield among the islands. The pair-wise multiple comparisons mean tests revealed that cattle from Maui Island had significantly lower carcass weight (*p < 0.05*) than those from other neighboring islands (Figure 2D; Supplementary Tables S3, S4), whereas the carcass weight of cattle from the other three islands did not differ significantly.

### Population structure and linkage disequilibrium decay

**FIGURE 3:**
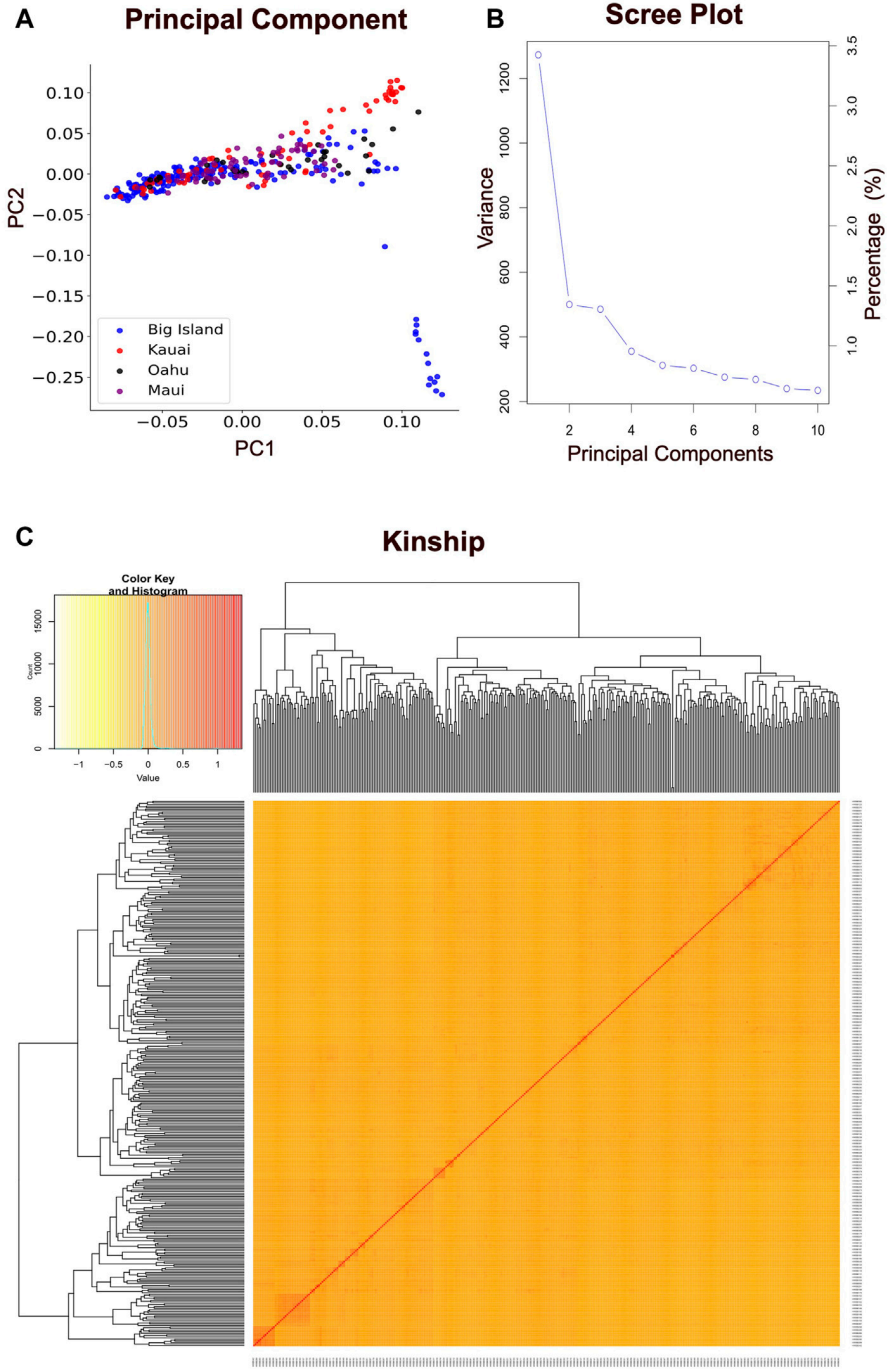
Population structure analysis. **(A)** The principal component analysis (PCA) results for 351 Hawai’i cattle from four islands, using 85K quality-controlled SNP data. Each colored dot represents an individual animal located on a different island. The horizontal and vertical axes represent the first and second principal components, respectively, contributing to 3.5% and 1.5% of the total variability in the data. The PCA analysis provides insight into the genetic relationships among cattle populations and identifies patterns of genetic variation. **(B)** The scree plot illustrates the variance accumulation of the top ten principal components (PCs). The *x*-axis represents the top ten PCs, while the *y*-axis represents eigenvalues that signify the amount of variation. The accumulated variance for each PC is denoted by an empty circle. The “elbow” point of the curve, where the slope begins to level off, signifies the number of factors that the analysis should generate. **(C)** The hierarchical clustering and heat map of the pairwise kinship matrix values, calculated based on 85K quality-controlled SNPs from 351 Hawaiian cattle. The color histogram illustrates the distribution of the coefficient of coancestry, with stronger red colors indicating higher levels of relatedness among individuals.

**FIGURE 4:**
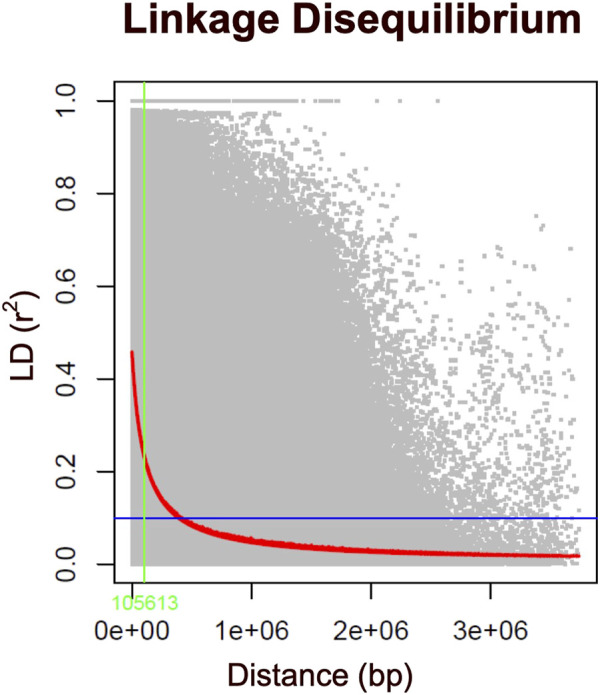
Genome-wide linkage disequilibrium (LD) decay plot for 351 Hawaiian cattle based on 85K SNP markers. The LD, measured as the squared correlation coefficient (r^2^) between pairs of polymorphic markers, is plotted against their genetic distance (bp) across the chromosomes. The red line represents the moving average of the 10 adjacent markers, while each gray dot represents a pair of distances between two markers on the window and their corresponding squared correlation coefficient. The blue line denotes the LD cutoff of 0.1, and the green line indicates the critical LD at a distance of less than 100 kb.

The present study employed principal component analysis (PCA) to investigate the genetic structure of 351 Hawai’i cattle, utilizing quality-controlled single nucleotide polymorphism (SNP) data. The analysis revealed that there were no distinct genetic clusters among the sampled accessions, as shown in Figure 3A. This observation is consistent with a uniform genetic background of cattle across the Island chain, leading to the presence of a single linear cluster with no population structure. Furthermore, the results demonstrated that the cattle herds across the Hawai’i island chain shared similarities, with no distinct clusters based on allelic SNPs explained by the first two principal components. The scree plot for eigenvalues revealed that the first two principal components explained 5% of the total variability. The sharply declined elbow at the second PC indicates that the first PC was the major source of variability used to correct for possible population structure, while subsequent PCs explained less variability after reaching the lowest elbow point (Figure 3B). Additionally, the heatmap and dendrogram of the kinship matrix confirmed the absence of clear clusters in the population, indicating that the cattle population in this study is unrelated by family (Figure 3C). The square of the correlation coefficient between the markers at two loci (*r* ^2^) was used to evaluate the LD (linkage disequilibrium) estimate. When LD reaches an *r* ^2^ value below 0.2, it is typically expected to decay by half (Vos et al., 2017; Singh et al., 2021). In the case of the Hawai’i cattle population under investigation, the LD decay reached an *r* ^2^ value of 0.15 at approximately 100 kb (Figure 4). Thus, to identify candidate genes associated with the carcass weight of beef cattle, we searched for genes within a 100 kb range upstream and downstream of the identified SNPs using the UCSC genome browser.

### Association analysis

**FIGURE 5:**
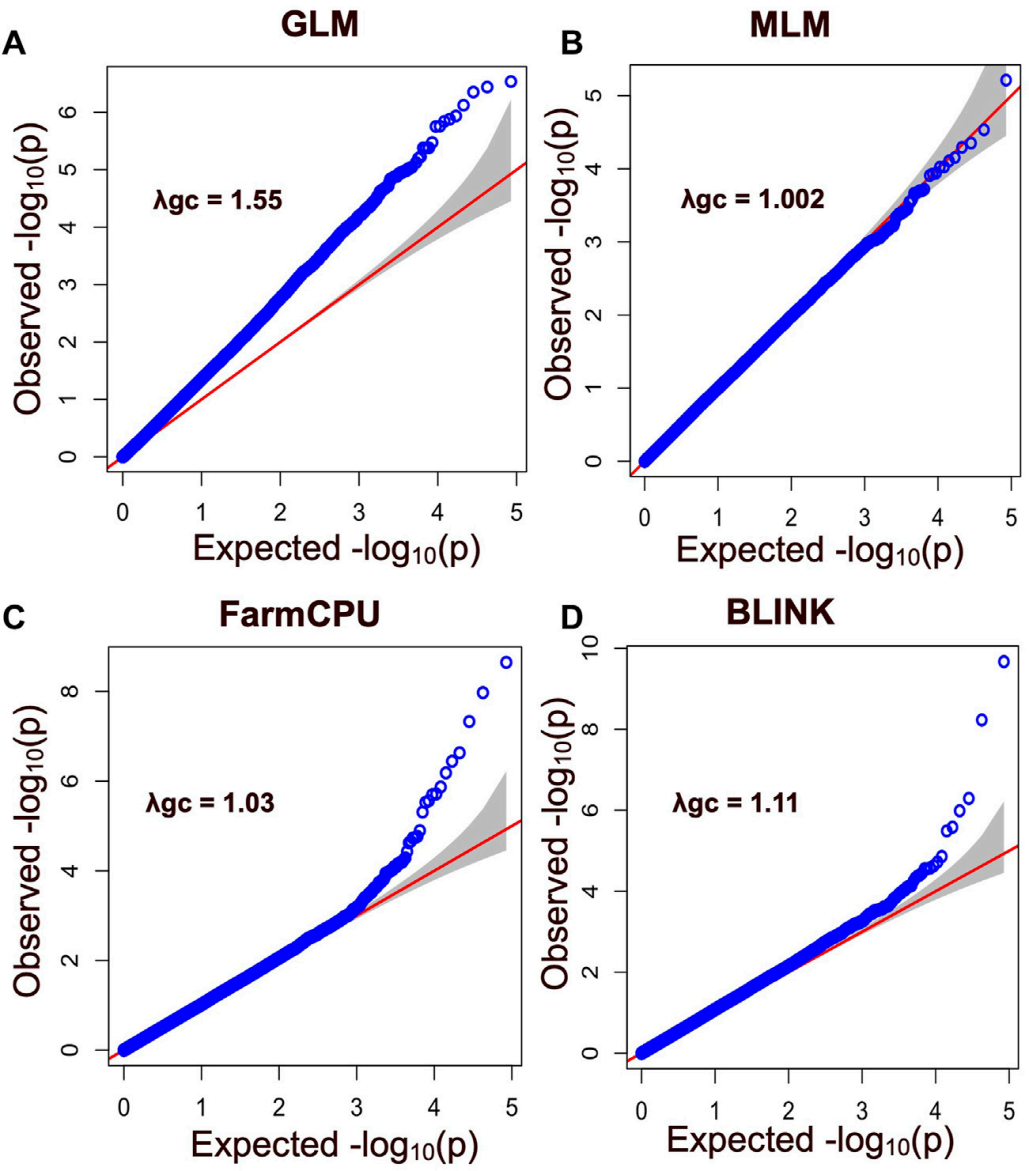
Quantile - Quantile (QQ) plot showing SNP markers with their observed and expected *p values* for different models including **(A)** General Linear Model (GLM), **(B)** the Mixed Linear Model (MLM), **(C)** the Fixed and Random Model Circulating Probability Unification (FarmCPU), and **(D)** the Bayesian-Information and Linkage-Disequilibrium Iteratively Nested Keyway (BLINK). Blue circles correspond to the *p*-values derived from the principal components + kinship model, while the red line indicates the expected *p*-value distribution under the null hypothesis that the *p*-values follow a uniform [0, 1] distribution. The gray shadow area represents the 95% confidence interval for the QQ plot under the null hypothesis of no association between the SNP and the trait. The -log 10 (p) negative base 10 logarithms of the *p*-values (probability of type-I error made in GWAS hypotheses testing) are also shown. This plot provides insight into the distribution of significant associations between the SNP and the trait, with deviations from the expected distribution indicating the presence of false-positive associations or other factors affecting the association analysis.

**FIGURE 6:**
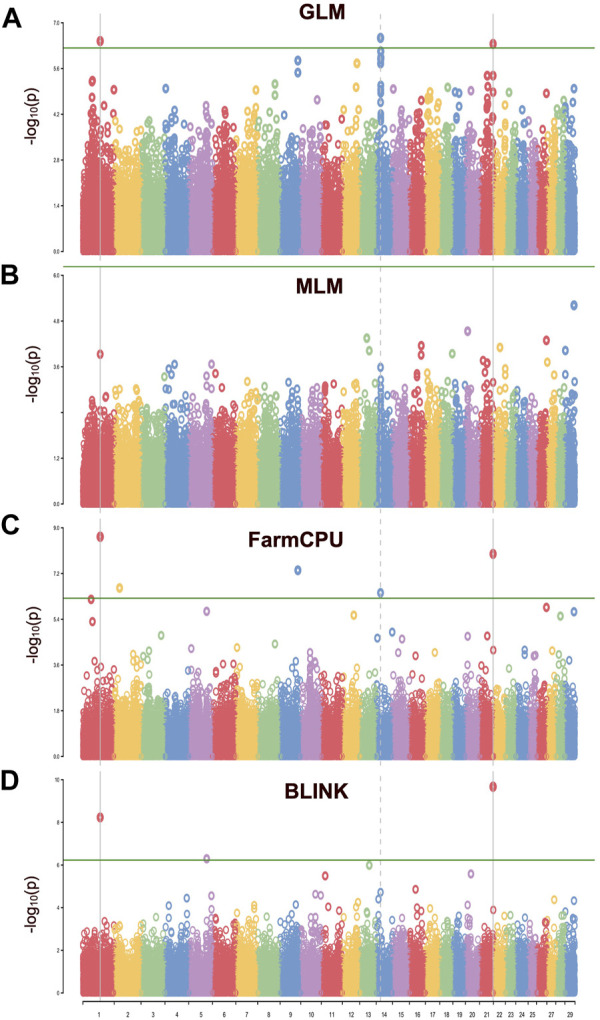
The Manhattan plot for the GWAS of carcass weight in the Hawaiian cattle population. Each chromosome (*x*-axis) is represented by a different color, and the plot is based on -log 10 (*p*-value) from GWAS against chromosome position. The green line indicates the genome-wide significant threshold at *p* = 10e-6, while vertical lines highlight the chromosomes containing significant SNPs. **(A)** SNPs identified by single-locus GLM. **(B)** No SNPs were identified by single-locus MLM. **(C)** SNPs identified by multi-locus FarmCPU model **(D)** SNPs identified by multi-locus BLINK model. This plot provides an overview of the distribution of significant associations across the genome, with peaks indicating regions of potential interest for further investigation.

In this study, four different models were used to compare their strengths in controlling false positives and false negatives in the population. Among them, two multi-locus models, BLINK and FarmCPU, coincided with the expected straight line diagonally with a sharp deviation at the tail, indicating true association by controlling both false positives and false negative markers. The genomic inflation factors (λgc) were calculated for four models to check our population stratification, where GLM had a λgc of 1.55, MLM had a λgc of 1.002, FarmCPU had a λgc of 1.03, and BLINK had a λgc of 1.11 (Figure 5). The quantile-quantile (qq) plot showed a significant upward deviation from the straight line in GLM, indicating a high incidence of false positives. Conversely, the qq plots for the MLM, FarmCPU, and BLINK models exhibited a normal distribution of *p*-values, with λgc values close to 1, indicating effective control of spurious results and a high likelihood of true associations. Among the four models used in the association study, three models (GLM, FarmCPU, and BLINK) identified significant SNPs associated with carcass weight. Five significant SNPs were identified with the FarmCPU model while the other three significant SNPs were identified with the model BLINK (*p < 10* ^*−6*^) (Figures 5, 6). Two of these SNPs, “*BTA-40510-no-rs*” and “*BovineHD2100020346*”, were shared between the two models and are considered to have a stronger association with carcass weight in Hawai’i beef cattle herds (Table 1). Additionally, a few SNP markers are unique with each model: “*BovineHD0100011931*”, and “*BovineHD0200007999*” on chromosomes 1 and 2 were found with FarmCPU solely, and “*BovineHD0500025848*” on chromosome 5 was found only with BLINK. Three SNP markers identified with GLM are shared with other models. Without validation from other more robust models, results from GLM alone would have been inconclusive. However, the presence of the same markers in multi-locus models erased the doubt for false positives by GLM. The MLM was too conservative and was not able to identify any associated markers for carcass weight (Figures 5, 6). False negatives may have arisen due to model overfitting, as this population was free from population structure and family relatedness. In addition, adjusting the *p*-value threshold to False Discovery Rate (FDR <0.05) resulted in the identification of a greater number of significant SNPs. Specifically, 58 significant SNPs were identified after FDR adjustment, and these are listed in Supplementary Table S2.

**TABLE 1:** List of the significant SNPs (*p < 10* ^*−6*^) associated with carcass weight in the Hawaiian beef cattle population.

SNP	Position	MAF	Allele	Effect	Model	Candidate genes
BTA-40510-no-rs	chr1: 88012422	0.45441595	G/A	0.054	FarmCPU, BLINK, GLM	*ZMAT3*
BovineHD0200007999	chr2: 27466243	0.36324786	C/T	−0.045	FarmCPU	*CERS6*
BovineHD0500025848	chr5: 90643657	0.16524217	A/C	0.052	BLINK	*PLEKHA5*
BTB-01839335	chr9: 89664367	0.48005698	A/G	−0.043	FarmCPU	*MYCT1*
BovineHD1400006853	chr14: 21949250	0.09116809	T/C	0.084	FarmCPU, GLM	*RGS20, TCEA1, LYPLA1, MRPL15*
BovineHD2100020346	chr21: 68056605	0.43589744	A/C	0.047	FarmCPU, BLINK, GLM	*EIF5, CKB, MARK3*

Notes: MAF, minor allele frequency; Allele: The first allele is the nucleotide of the reference allele; The second allele is the nucleotide of the alternate allele; Effect: the contributing weightage of SNPs to carcass weight; Model, the different models that successfully identified SNPs associated with carcass weight; Candidate Genes, the genes that correspond to the significant SNPs in the range of upstream 100 kb and downstream 100 kb (reference genome, ARS-UCD1.2/bosTau9).

### Identification of candidate genes

All the significant SNPs identified in our population were explored for their biological function annotation. Genes within 100 kb upstream and downstream relative to the identified SNPs were scanned, and eleven genes (*ZMAT3, CERS6, PLEKHA5, MYCT1, RGS20, TCEA1, LYPLA1, MRPL15, EIF5, CKB, and MARK3*) were identified to be associated with carcass weight in Hawai’i cattle. Also, eight of these genes (*ZMAT3, RGS20, TCEA1, LYPLA1, MRPL15, EIF5, CKB, and MARK3*) overlapped with significant SNPs identified by at least two models (FarmCPU, BLINK, and GLM) (Table 1). Details of candidate genes (Gene name, Chromosome number, Ensemble ID) and their biological functions in other mammals including humans, mice, sheep, pigs, and dairy cows are presented in Supplementary Table S5. It is worth mentioning that five of these genes (*RGS20, TCEA1, LYPLA1, MRPL15*, and *EIF5*) have been previously identified in several studies to be associated with carcass traits and growth traits in cattle (Lindholm-Perry et al., 2012; Hay and Roberts, 2018; Doyle et al., 2020). *ZMAT3* has been reported to be correlated to conception rate and fertility in Brangus cattle (Fortes et al., 2012), and *CKB* and *MARK3* are related to milk production and somatic cell score in Holstein dairy cattle (Buaban et al., 2022). *PLEKHA5*, *MYCT1*, and *CERS6* were identified for the first time in cattle in our study, however, these genes are conserved in more than 200 other organisms, vertebrates, and mammals, including humans, chimpanzees, rhesus monkeys, dogs, mice, rats, and cows (https://www.ncbi.nlm.nih.gov/gene/). The specific roles of these genes in cattle have not been defined yet and may serve as supporting and maintenance functions.

## Discussion

### Association mapping and efficient model for GWAS in cattle

Out of the four models used in this study, BLINK, FarmCPU, and GLM performed well in predicting significant SNP markers for carcass weight, while MLM failed to identify the markers with the same trait. The GLM commonly produced false positives and the MLM commonly produced false negatives. These results were consistent with other published results (Tamba et al., 2017; Wen et al., 2018). Based on the results from this study, it is possible to conclude that, a conventional single-locus model like MLM was too stringent to identify the SNP markers. MLM can produce better results when there is evidence of population structure due to the geography diversity or family relatedness, but the cattle in this study were from the same geographic area (Hawai’i Pacific) and represented several farms, ruling out the alleles to have family relatedness, which was reflected in our results of no kinship clusters observed (Figure 3C). However, weaker family relatedness observed as small patches across the diagonals at multiple spots in the kinship heat map plot could not be ignored, and therefore, the kinship matrix was fitted as covariates to adjust the confounding effect due to family relatedness in our model. MLM accounts for both covariates due to PC and kinship; therefore, the model got overfitted and might have resulted in false negative SNPs. In contrast, QQ plots with the FarmCPU and BLINK models showed a straight line close to the 1:1 with a slightly deviated tail, indicating that FarmCPU and BLINK controlled false positives and false negatives without compromising the results for associated markers (Figure 5). Our main results i.e., identified SNPs were primarily from FarmCPU and BLINK, while GLM identified the same markers as did by the other two models. The number of PCs seems to affect less multilocus models such as FarmCPU and there is no concrete gold standard for how many PCs to be included to correct for the possible population stratification (Wang and Zhang, 2021). We followed a general convention of using the number of PC-based observations of the elbow on the scree plot. Our results had an elbow on the second PC, indicating the first PC is the major source of variability. Therefore, we used a single PC to correct for population stratification, which is almost equivalent to the results of using a sole kinship model with no PCs as covariates. However, including one PC helped to elaborate the model making it a full model for GWAS. Further, the genomic inflation factor (λgc) ranged between 1.002 and 1.11 among MLM, FarmCPU, and BLINK, indicating that these three models best fitted for GWAS in Hawaiian cattle herds, while GLM did not fit properly with λgc above 1.5. Results from GLM would have been questionable, as the QQ plot deviated sharply from the expected line (λgc > 1.1). However, three shared SNP markers identified by multi-locus models increase the validity of the true association between SNP markers and the trait of carcass weight in our study, which is similar to a single-trait GWAS study in wheat, FarmCPU and BLINK performed better than MLM in identifying the associated markers (Merrick et al., 2022).

In this study, FarmCPU identified five significant markers, which are more than BLINK identified; two of those significant SNP markers are common in both models. Researchers are increasingly using multi-locus models in association mapping, more exclusively in plants and some in animal studies. In a similar study in plants, researchers compared several qualitative traits in soybean and maize flowers using eight popular models where FarmCPU performed better for most of the traits than other models, including GLM and MLM (Kaler et al., 2020). FarmCPU is gaining popularity today due to improved statistical power when compared to other methods. The problem of model overfitting is minimized when using the FarmCPU model because of a two-step adjustment. The first adjustment involves fitting the covariates from population structure, family relatedness, and pseudo-quantitative trait nucleotides. The second adjustment involves either refining the covariates or selective inclusion or exclusion of pseudo-quantitative traits based on their relationship with the testing markers (Liu et al., 2016). Therefore, model selection becomes a crucial step in GWAS to prevent the loss of valuable markers as false negatives and to control the biased associations that are not truly associated with the traits and appear as false positives. In addition, single-locus models, which only consider a one-to-one independent relationship between markers and traits, do not accurately apply to the biological phenomena as the interaction of genes is a common phenomenon in trait expression. In contrast, multi-locus models simultaneously test the association of multiple markers for a given trait (Liu et al., 2016), which is closer to biological phenomena involving gene action and interactions. MLM initially gained popularity over GLM due to its higher statistical power, however, multi-locus models like FarmCPU and BLINK surpass both GLM and MLM in statistical power and computational efficiency (Liu et al., 2016; Huang et al., 2019). MLM only considers population structure and kinship as covariates so markers in various loci that are not significant sometimes may appear as false positives or false negatives. In contrast, FarmCPU establishes a relationship with the marker at one locus and treats all other markers at different loci as covariates, reiterating again and again and completing the K iteration, where K = SNPs ((Liu et al., 2016). This way assigning the non-significant markers at multiple loci as covariates minimizes the chances of their appearance as false positives or false negatives with multi-locus models (Liu et al., 2016; Tang et al., 2019; Kaler et al., 2020), which was also observed in our results where FarmCPU and BLINK outperformed over MlM. Based on our results, it can be suggested that using multi-locus models for association mapping in animal-modeled research studies may be a better option than relying solely on single-locus models, as is commonly done in recent association studies in plant models.

### Candidate genes related to carcass traits in cattle

Bovine chromosome 14 (BTA14) has been widely explored for quantitative trait loci (QTL) and genes related to feed intake, weight gain, and carcass traits in dairy and beef cattle (Smith et al., 2019; Srikanth et al., 2020). The genes, located in a conserved region on BTA14, have been reported as a selective sweep region in dairy and beef cattle breeds (Zhao et al., 2015), and the DNA regions on BTA14 have been associated with backfat thickness, rib eye muscle area, marbling, and other carcass traits in beef cattle (Lindholm-Perry et al., 2012; S. H. Lee et al., 2013; Zhang et al., 2019). In another recent study, *RGS20, TCEA1, LYPLA1*, and *MRPL15* on BTA14 have been associated with the back fat thickness (BFT) and Intra Muscular Fat (IMF) in a composite beef cattle breed (Hay and Roberts, 2018). *RGS20* has also been found to be involved in actin cytoskeleton organization which governs meat tenderness in European beef cattle breeds (Mengistie et al., 2022). *TCEA1* and *LYPLA1* have been associated with average daily feed intake and average daily weight gain in composite cattle breeds (Lindholm-Perry et al., 2012; Grigoletto et al., 2019; Hay et al., 2022). Furthermore, *RGS20* was associated with thigh width in Angus breeds (Doyle et al., 2020) and average daily weight gain in Yorkshire pig breeds (Cai et al., 2022). *EIF5* gene on chromosome 21 was also found to be associated with marbling and carcass traits in Nellore cattle (Carvalho et al., 2019). Expression of the *EIF5* gene positively contributes to the growth of the longissimus thoracis muscle in *Bos. Indicus* (Bruscadin et al., 2021). Candidate genes identified in this study and their roles in some other breeds of discussed above presented in Table 2. From these findings and discussions, it can be concluded that *RGS20, TCEA1, LYPLA1, MRPL15*, and *EIF5* genes are strongly associated with carcass weight in Hawai’i beef cattle. Additionally, *MYCT1* and *CERS6* are possibly candidate genes for carcass weight in cattle, as their roles have been identified in pigs and sheep (Wu et al., 2020; Xu et al., 2021). Further studies are required to ascertain the association of *MYCT1* and *CERS6* genes with carcass weight and to better understand their biological roles in cattle.

**TABLE 2:** Candidate genes and their roles in beef cattle.

Candidate gene	Role in cattle	Literature
*RGS20*	Back fat thickness, Intramuscular fat, and meat tenderness in composite beef cattle breeds	Hay and Roberts (2018)
*TCEA1*	Growth traits in Montana tropical composite cattle	Grigoletto et al. (2019)
*LYPLA1*	Feed intake, growth, and average daily weight gain in composite beef cattle and cross breeds	Lindholm-Perry et al. (2012); Hay et al. (2022)
*MRPL15*	Residual feed intake in Australian Angus; Muscle growth in cattle	Cassar-Malek et al. (2007); Heras-Saldana et al. (2019)
*EIF5*	Muscle growth, marbling, and meat quality traits in Nellore cattle	Carvalho et al. (2019); Bruscadin et al. (2021)

### Candidate genes in other mammals

Previous work identified *CERS6* and *MYCT1* genes have an association with carcass-related traits in other animals (Wu et al., 2020; Xu et al., 2021; Buaban et al., 2022) but not in beef cattle. Few of the genes (*ZMAT3*, *PLEKHA5, CKB,* and *MARK3)* identified in our study were reported in dairy cattle (Buaban et al., 2022). Very little information on above mentioned six genes within beef cattle is available, however, some details about their biological processes and homologs in other mammals including humans, mice, sheep, pigs, and dairy cows are listed in Supplementary Table S5. *CERS6* and *MYCT1* have been studied to be associated with obesity, weight gain, and subcutaneous fats in several mammalian species, including humans, mice, sheep, and pigs. *CERS6* enables sphingosine N-acyltransferase activity and is involved in the membrane’s ceramide biosynthetic process. In an association study, *CSER6* was associated with fat deposition in sheep (Xu et al., 2021). Another study found that *CERS6* was associated with subcutaneous fat in lamb in response to a concentrate-supplemented diet (González-Calvo et al., 2017). *CERS6* expression positively correlates with BMI, body fat content, and obesity in humans. Upregulation of *CERS6* and subsequent increase in specific acyl-chain ceramides contributes to both murine and human obesity (Turpin et al., 2014). *MYCT1* gene was predicted to regulate specific *MYC* target genes. The role of the *MYCT1* gene in cattle has yet to be studied, but it is associated with meat quality and pH value in Qingyu pigs, specifically, *MYCT1* is involved in skeletal muscle development, regulation of Ca^2+^ release in the muscle, and anaerobic respiration, governing superior meat quality traits in Qingyu pigs (Wu et al., 2020). These genes (*CERS6* and *MYCT1*) are mostly related to muscles, subcutaneous fat, and obesity in humans, mice, sheep, and pig’s meat, placing them in the list of possible candidate genes for carcass-related traits in beef cattle. CKB (Creatine Kinase B) is conserved in 315 mammals, including humans, mice, monkeys, and cattle. A previous study revealed that the *CKB* gene located in BTA 21 governs the fertility of cattle (Han and Peñagaricano, 2016). The *CKB* gene encodes an enzyme creatine kinase, and the elevated level of creatine kinase in the sperm causes oligospermia and male sterility (Gergely et al., 1999). *MARK3* is associated with bone mineral density in humans and mice (Calabrese et al., 2017). The protein encoded by this gene is activated by phosphorylation and in turn, is involved in the phosphorylation of tau proteins MAP2 and MAP4. *PLEKHA5* has been associated with milk-fat yield in Holstein cattle (Jiang et al., 2019; Pedrosa et al., 2021). *CKB* and *MARK3* genes located together in the same genomic region on chr21 have been reported to be associated with milk production and somatic cell score in Holstein cattle (Buaban et al., 2022). Therefore, genes *PLEKHA5, CKB,* and *MARK3* identified from our study might indirectly contribute to weight gain and carcass traits during the early stage of growth under cow-calf operation, ensuring optimum milk supply before weaning age. However, there is no clear evidence yet for these genes (*ZMAT3*, *CERS6, PLEKHA5, MYCT1, MARK3*, and *CKB*) regarding their association with carcass-related traits in beef cattle, and further research on their functional validations is required to confirm whether these genes are indeed associated with carcass weight and meat quality traits in beef cattle.

## Conclusion

Multi-locus models such as FarmCPU and BLINK were found to be superior to single-locus models (GLM and MLM) in identifying SNP markers and minimizing false positives. Two SNP markers were identified using both multi-locus models. Three other markers were identified using GLM and FarmCPU models, strengthening the correlation of these SNPs with carcass weight in beef cattle. The *EIF5* gene on chromosome 21 and four other genes in the BTA14 region (*RGS20, TCEA1, LYPLA1,* and *MRPL15*) were found to be associated with carcass weight in Hawai’i beef cattle, and these results align with the previous findings showing their correlation with carcass weight and related traits in other cattle breeds. Future work incorporating selection pressures using these genes (*EIF5, RGS20, TCEA1, LYPLA1,* and *MRPL15*) will facilitate genetic improvement in Hawaiian beef cattle, enhancing productivity while utilizing limited resources without harming the delicate ecosystem of the island.

## Data Availability

Our SNP genotyping data reported are available in the DDBJ Genomic Expression Archive under the accession number PRJDB15706.
